# Reliability generalization meta-analysis of the Climate Change Worry Scale

**DOI:** 10.3389/fpsyg.2025.1590126

**Published:** 2025-04-25

**Authors:** Melehat Gezer, Yıldız Yıldırım, Mustafa İlhan

**Affiliations:** ^1^Faculty of Education, Social Studies Education, Dicle University, Diyarbakir, Türkiye; ^2^Faculty of Education, Measurement and Evaluation in Education, Aydin Adnan Menderes University, Aydin, Türkiye; ^3^Faculty of Education, Department of Mathematics and Science Education, Dicle University, Diyarbakir, Türkiye

**Keywords:** Climate Change Worry Scale, internal consistency, meta-analysis, reliability generalization, reliability induction

## Abstract

Climate change worry is an increasingly critical issue in eco-psychology literature. A commonly used instrument for measuring this construct is the Climate Change Worry Scale (CCWS), developed by Stewart. This Likert-type scale assesses individuals' climate change worry through 10 items clustered under a single factor. It has been adapted for multiple cultures and utilized in numerous studies conducted across various countries. Nevertheless, no study has synthesized the reliability values obtained from individual studies for the scale. The purpose of the current meta-analysis was to perform a reliability generalization for the CCWS. To this end, an exhaustive literature search was conducted from July 14 to November 17, 2024, in the EBSCO, ERIC, Taylor & Francis, PubMed, and Web of Science databases, as well as Google Scholar, using the keyword “Climate Change Worry Scale.” After scrutinizing the identified studies for duplicates and applying inclusion and exclusion criteria, the research focused on the 40 Cronbach's alpha coefficients acquired from 37 papers. The results of the analysis, which involved running the random effects model and the Bonnet transformation, indicated that the pooled Cronbach's alpha was 0.932 (95% CI = 0.919–0.942). The results of the moderator analysis revealed that the sample descriptors and study characteristics included in the meta-analysis did not significantly affect the reliability estimates. Accordingly, the CCWS was found to be an instrument that produces highly reliable measurements regardless of factors such as region, language, participants' age, and the total number of items answered during administration. Finally, the reliability induction rate was determined to be 29.41%. However, the high heterogeneity observed among the reliability estimates of the primary studies exposed the limitations of generalizing the reliability of CCWS scores across different populations and research conditions. This situation also emphasized the importance of providing detailed information about the scale's sample demographics and administration conditions when reporting reliability.

## Introduction

Climate change is considered the major and most widespread challenge that the natural environment and humanity have ever faced (United Nations, 2022). Its effects on both ecosystems and humans make the issue even more important. This concept is defined as statistically significant changes in the average condition of the climate lasting for decades or a more extended period (Türkeş, 2008). The climate is constantly changing due to many different natural factors. However, a crucial new factor that has been affecting the Earth's climate more and more in the last two centuries is human activity (Mikhaylov et al., 2020). Human-induced climate change, known as anthropogenic influences, causes many weather and climate extremes in almost every geographical area of the world (Trenberth, 2018). The human impact on extreme changes observed in the atmosphere, ocean, and land, such as warming, heatwaves, glacier loss, heavy rainfall, droughts, and tropical cyclones, has been demonstrated with evidence (IPCC., 2023).

The potentially devastating influences of climate change and its accompanying extreme weather events on people, the environment, and the economy have been widely documented (Sümen et al., 2025). For example, alterations in climate have provoked migrations in many locations from Africa to Asia and from North America to Central and South America (IPCC., 2023), to the extent that the term “climate refugees” has entered the literature due to these migrations (Lister, 2014). In addition to the notable economic losses identified in climate-sensitive sectors such as agriculture, forestry, fisheries, shellfish aquaculture, energy, and tourism in our times (IPCC., 2023), climate-related natural disasters have incurred substantial costs for countries (European Environment Agency, 2024). Moreover, numerous species are at risk of extinction due to climate change (McElwee, 2021), posing a potential threat to the Earth's biodiversity (Kappelle et al., 1999).

### The impacts of climate change on physical and mental health

Increases in extreme temperatures trigger various physical illnesses in humans, such as heat stroke, hypothermia, and diarrhea, as well as cardiovascular, respiratory, infectious, and pathogenic diseases (Avan and Vural, 2025; Comrie, 2007; D'Amato et al., 2014; Franchini and Mannucci, 2015; Kim et al., 2014), and can even result in fatalities (Arnold, 2022). Additionally, climate change may lead to a range of adverse effects related to mental health, including psychological distress, anxiety disorders, worry, acute stress, depression, aggression, insomnia, suicidal ideation, and a diminished sense of self and identity (Charlson et al., 2021; DeJarnett et al., 2025; Kumar et al., 2024; Palinkas and Wong, 2020). In a large-scale cross-cultural study by Hickman et al. (2021) involving over 10,000 participants aged 16–25 years, it was found that 84% of the respondents are at least moderately concerned about climate change. Furthermore, more than 45% of the participants reported that their feelings regarding climate change negatively impacted their daily functioning. It is anticipated that the negative effects of climate change on mental health will become increasingly pronounced in the future as climate-related stressors affect a larger number of people (Taylor, 2020). The impact of environmental degradation on emotional responses has also been highlighted in the eco-psychology literature, introducing new climate-specific mental health phenomena such as solastalgia [a state of feeling lonely, insecure, and powerless caused by significant changes in one's immediate environment due to the acute effects of climate change, including sea level rise, flooding, and forest fires] (Albrecht et al., 2007), ecological grief (Cunsolo and Ellis, 2018), eco-guilt (Cianconi et al., 2020), eco-anxiety, climate change anxiety (Albrecht, 2011; Clayton, 2020), and climate change worry (Stewart, 2021).

### Climate change worry

The number of studies on the psychological effects of climate change is rapidly increasing. Various concepts have been proposed in these studies to describe individual reactions to climate change. Anxiety and worry are two concepts frequently encountered in the literature on this subject. Clayton (2020) explained the effects of climate change on mental health by focusing on the concept of anxiety (a psychological state that can lead to restlessness and sleep disturbances), while Stewart (2021) focused on worry (verbal-linguistic thoughts about potential alterations in the climate system and their possible consequences). Although these two concepts are related, they are not identical, and it is incorrect to use them interchangeably (Innocenti et al., 2022). Anxiety is a broader construct expressed through affective (such as nervousness and fear), cognitive (difficulty concentrating), physiological (nausea and sweating), and behavioral (sleep disturbances, dysfunction) indicators (van Valkengoed et al., 2023). In contrast, worry pertains specifically to cognitive content, particularly excessive concern regarding future events (Ojala et al., 2021; Zebb and Beck, 1998).

Climate change worry is rooted in the perceived threat of climate change; a greater perceived threat can drive more worry (Reser and Swim, 2011). Worry about climate change forms the initial stage of all emotions related to this issue and has the potential to regulate emotions (Kurt and Akdur, 2024), serving as a motivating factor for individuals to take action on the matter (Bouman et al., 2020) and to engage in pro-environmental behaviors such as recycling and reducing their ecological footprint (Donati et al., 2025; Kabasakal Çetin et al., 2025). If this worry becomes maladaptive and chronic in an individual's life, particularly with increases in autonomic arousal (like elevated heart rate, respiration, sweating, and tension), it can lead to climate anxiety (Innocenti et al., 2022; Stewart et al., 2024). Worry also relates to perceived risk and fear, yet it is less cognitive than perceived risk and less overwhelming than fear. Climate change worry reflects an individual's active emotional engagement with the issue and personal concern regarding its consequences (Bouman et al., 2020). As long as climate change worry remains adaptive and does not become pathological, it enables individuals to pay attention to ongoing climate events, making it a healthier response than denial or disavowal (Dodds, 2021). In other words, while severe worry related to the topic can be extremely debilitating (Whitmarsh et al., 2022), milder worry is a rational and potentially functional reaction to the awareness of the serious threat posed to the planet (Martin et al., 2024). All these points highlight the importance of investigating climate change worry and assessing its level.

### Climate Change Worry Scale

Despite the increasing attention to the psychological effects of climate change, the number of valid and reliable instruments addressing these phenomena is relatively scarce. Studies generally use items and inventories designed for other applications (Cruz and High, 2022). The Climate Change Anxiety Scale (CCAS) (Clayton and Karazsia, 2020), the Climate Change Worry Scale (CCWS) (Stewart, 2021), and the Hogg Eco-Anxiety Scale (HEAS) (Hogg et al., 2021) are among the scales most frequently used by researchers. Of the mentioned scales, CCAS and HEAS are widely utilized to measure anxiety, while CCWS is employed to assess climate change worry. This study focused on CCWS rather than CCAS and HEAS, considering that while CCAS and HEAS are valuable tools, they prioritize emotional and anxious responses to climate change and may not capture cognitive aspects as precisely as CCWS. One reason for conducting the current RG meta-analysis on CCWS instead of CCAS was its generally higher predictive value in healthy, general samples (Plohl et al., 2023). Other reasons for choosing CCWS over CCAS and HEAS include its ease of application due to its brevity, likely ensuring more accurate and complete responses by reducing participant burden and possible respondent fatigue, streamlining data processing and analysis, thereby being a practical option even for large-scale studies.

The CCWS, developed by Stewart (2021) in English, has been adapted into multiple languages and cultures, including Turkish, Spanish, Polish, French, and Hebrew. Despite being recently developed, a total of 96 citations were found in Web of Science, and 218 results were identified in Google Scholar when the scale was searched in the literature. The original form of the CCWS consists of 10 items with a five-point Likert-type rating scale. It has a unidimensional structure that explains 73.60% of the total variance, with factor loadings of the items in this single-factor structure ranging from 0.73 to 0.90. In the original version of the scale, Cronbach's alpha and McDonald's omega coefficients were calculated to assess reliability, both showing internal consistency coefficients of 0.95 (Stewart, 2021). In addition to providing valid and reliable measurements, the CCWS's concise nature, consisting of only 10 items, makes it a valuable instrument for researchers studying climate change worry.

### The purpose and importance of the research

Numerous studies have utilized the CCWS across various geographical locations and cultures, involving different age groups and diverse sample sizes with varying gender distributions. The existing studies report differing reliability coefficients for the measurements obtained through the CCWS. For example, while there is a study that calculated a reliability value slightly above 0.70 for the data collected with the CCWS (Duran and Kaynak, 2024), there are also studies that reach reliability coefficients above 0.95 (e.g., Özbay and Alci, 2021; Plohl et al., 2023). Additionally, a review of conducted studies revealed instances of reliability induction (Vacha-Haase et al., 2000); that is, some researchers cited reliability values from prior studies related to the development or adaptation of the CCWS without providing the reliability of their own data. However, reliability is not a fixed attribute of the measurement tool; it varies according to sample characteristics, administration conditions, and other factors (Bandalos, 2018; Crocker and Algina, 1986). Therefore, researchers must not only report the reliability of the studies from which their scales were developed or adapted but also disclose the reliability of their own data. Given these considerations, it is essential to conduct a reliability generalization (RG) meta-analysis for the CCWS to obtain a pooled reliability coefficient based on the coefficients calculated across various cultures and samples and to investigate possible variables that may account for the heterogeneity in the reported reliability coefficients. In this context, the current study posed the following questions:

1) What is the overall Cronbach's alpha coefficient for the CCWS?
2) How do the sample descriptors and study characteristics (region, language scale, gender distribution, age, and the total number of items respondents answered during the administration of the CCWS) affect the pooled reliability coefficient?
3) What is the reliability induction rate in studies using the CCWS?

Based on the research questions outlined above, the current meta-analysis of individual empirical studies utilizing the CCWS will provide insights into the percentage of studies in which reliability was misinterpreted due to reliability induction. Furthermore, as Sen and Yörük (2023) noted, the presence of variables that may cause systematic errors in scale scores will be revealed through moderator analysis. Specifically, this study will enhance our understanding of whether errors in scale scores are random (related to unpredictable factors such as respondents' low motivation, inattention, etc.) or systematic, stemming from variables such as gender, culture, scale language, and the total number of items answered by respondents. In this regard, this investigation has the potential to provide more comprehensive information regarding the reliability of the CCWS compared to individual studies, allowing researchers to use the scale more effectively and interpret the reliability of its scores accurately. To date, numerous RG meta-analyses have been conducted on various measurement tools across different disciplines. A search for the keyword “reliability generalization meta-analysis” in Google Scholar on March 3, 2025, yielded 2,430 results. To the best of our knowledge, no effort has been made to consolidate the individual reliability evidence of the CCWS. Thus, the current meta-analysis represents an original study that could enhance the evidence regarding the psychometric properties of the CCWS. Since it was not possible to access factor loadings or inter-item correlation matrices from individual studies that employed the scale, except for the papers where the CCWS was developed or adapted, this meta-analysis focuses solely on RG, a meta-analytic factor analysis regarding the validity of the CCWS was not conducted. Given that reliability is a prerequisite for validity (Morrison et al., 2011), it is anticipated that this research will establish a foundation for future meta-analytic factor analysis studies on the CCWS. In this respect, the present study remains highly valuable even without a meta-analysis of validity.

## Method

The current RG meta-analysis was conducted by adhering to the REGEMA checklist developed by Sánchez-Meca et al. (2021), which is based on previous guidelines proposed in the field of meta-analysis.

### Data sources and literature search

A comprehensive literature search was conducted in the EBSCO, ERIC, Taylor & Francis, PubMed, and Web of Science databases using the keyword “Climate Change Worry Scale.” The search was performed from July 14, 2024, to November 17, 2024, without any restrictions on publication types. Since the CCWS was published in 2021, the relevant studies covered a period from 2021 to November 2024, inclusive. To ensure that no eligible studies were overlooked, the reference lists of retrieved studies were manually reviewed (backward search), and Google Scholar, identified as a gray literature repository by Jahrami et al. (2024), was also searched.

### Selection of the studies

The identified studies were reviewed based on the following inclusion and exclusion criteria. Empirical studies were included in the RG meta-analysis if they (1) administered the full 10-item version of the CCWS to participants, (2) reported the Cronbach's alpha coefficient of their data along with the sample size, and (3) were published in Turkish or English. Conversely, studies were excluded if they (1) were theoretical or qualitative studies, meta-analyses, or systematic reviews, (2) utilized a scale other than the CCWS, (3) used only partial CCWS items in their instrument, (4) did not report the sample size or Cronbach's alpha coefficient of the available data and did not respond to the authors' email request regarding this issue, and (5) were published in a language other than Turkish or English. In calculating the reliability induction rate, studies that met the first and third inclusion criteria but did not present either the Cronbach's alpha coefficient or any other reliability estimation for their data were considered papers that induced reliability (even if the Cronbach's alpha coefficient was provided in response to the authors' email request).

**Figure 1:**
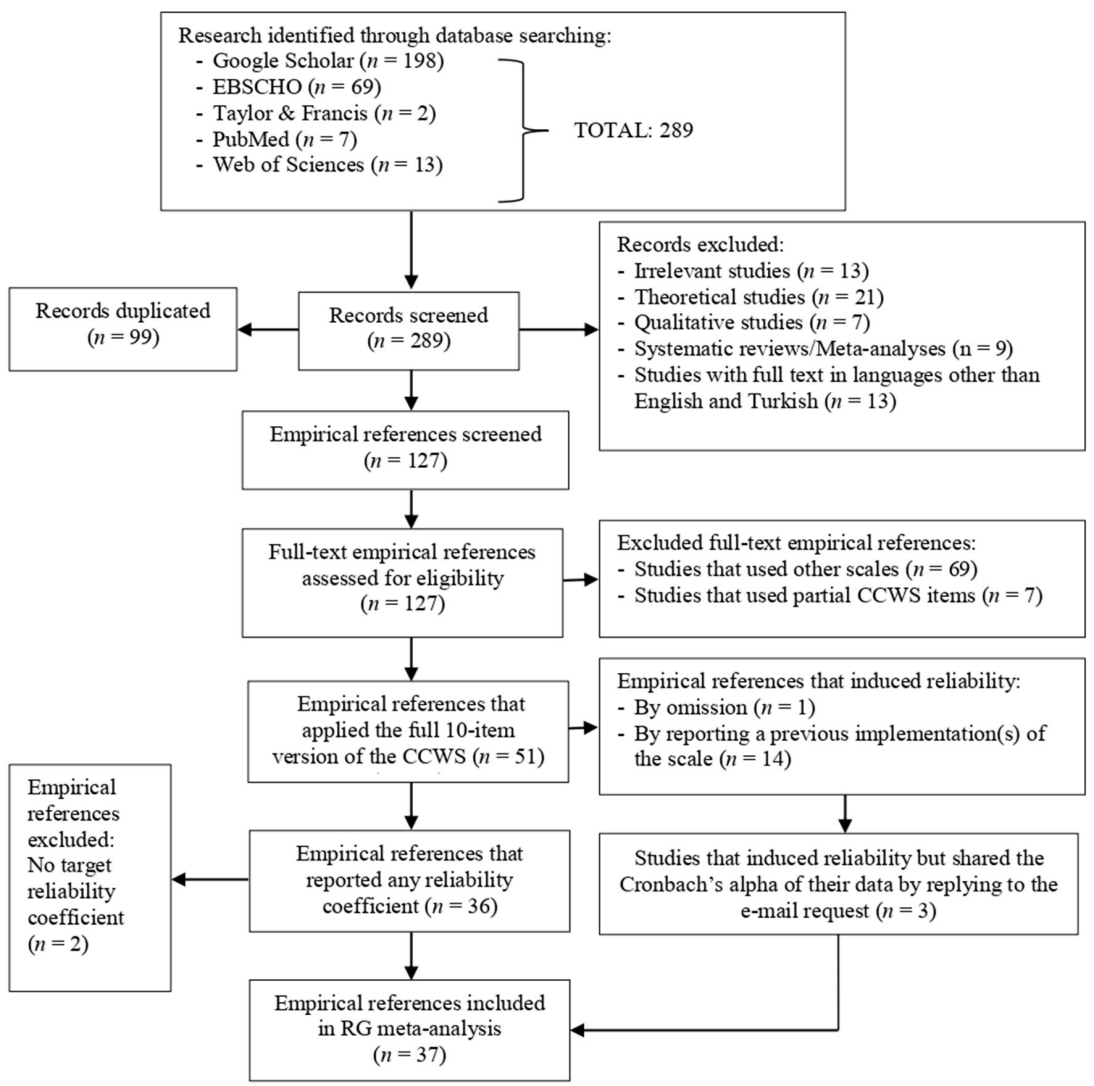
REGEMA flowchart for CCWS.

Figure 1 illustrates the flowchart summarizing the selection process, including both elimination and inclusion, of the studies incorporated in the RG meta-analysis. As shown in Figure 1, 289 references were initially identified, of which 99 were duplicates, and 63 were excluded for various reasons. When the full text of the remaining 127 references was reviewed, it was found that 69 employed a scale other than the CCWS, and 7 included only some of the CCWS items in their data collection tools. By excluding these 76 studies, the number of empirical references implementing the full 10-item version of the CCWS was reduced to 51. While one of the 51 studies indicated that it did not calculate a reliability coefficient due to its small sample size, the other 14 studies did not report any reliability estimates without explanation. For studies that applied the CCWS but did not provide Cronbach's alpha coefficient regarding scale data, an email was sent to the corresponding authors, leading to the acquisition of reliability estimations for three studies. Thus, the number of references implementing the CCWS and reporting reliability estimates based on the indexed sample increased from 36 to 39. However, since two of the 39 references provided a reliability estimate other than Cronbach's alpha, which is the target reliability index of this investigation, the RG meta-analysis was conducted on the 40 Cronbach's alpha estimates obtained from 37 studies.

### Data extraction

Initially, a coding schema was developed to extract relevant study characteristics and reliability coefficients. The extracted data included study metadata, scale language, sample characteristics (size, region, age, female ratio), reported reliability estimates, and the total number of items in the instrument applied to the participants. Pentapati et al. (2025) indicated that language and geographical location could influence the participants' definitions and perceptions of conceptual words in the scale items; therefore, these two variables were included among the moderator variables of this meta-analysis. The ratio of females to the total sample size was selected as a moderator because studies show that women have significantly higher climate worry scores than men (e.g., Clayton et al., 2023; Verplanken et al., 2020), suggesting that gender distribution may affect internal reliability by altering sample heterogeneity. Given the significant relationship between age and climate change anxiety, as well as perceived climate change threat (Searle and Gow, 2010; Whitmarsh, 2008), the sample's age (mean and standard deviation) was also included as a moderator variable. The reason for incorporating the variable of “total number of items” in the coding form was as follows: CCWS was administered alongside other scales in some studies, leading to variations in the number of items answered by participants from one study to another. It was thought that an increase in the number of responded items could degrade data quality due to factors such as fatigue and inattention, which could serve as a moderator variable generating heterogeneity in the reliability coefficient.

All 37 studies included in the meta-analysis were coded independently by two raters with double-blinding. One coder was an expert in social studies education, while the other specialized in measurement and evaluation. After the two researchers completed the coding, the reliability of the data extraction process was assessed. The agreement between coders was calculated using the formula proposed by Miles and Huberman (1994), resulting in 97.22%. This finding indicated a highly satisfactory consensus. Discrepancies between coders were primarily related to the variable of “total number of items” and were resolved through discussion. Table 1 summarizes the coding schema for the studies.

**Table 1:** Coding schema for the studies.

**Label**	**Type**	**Coding system**
Study tag	Categorical	The names of the author(s) and the year of publication
Title	Categorical	The title of the publication
Publication type	Categorical	Article, thesis, or proceeding.
Scale language	Categorical	Language of the scale (English, Turkish, Italian, French, etc.)
Country	Categorical	The country where the study was carried out (United States, Türkiye, Italy, France, Poland, Israel, etc.)
Sample size	Continuous	The number of participants in the dataset from which the reliability was estimated
Number of items	Continuous	The number of items on which the reliability of CCWS was calculated
Total number of items	Continuous	Total number of items in the instruments applied to the sample, excluding sociodemographic questions and control items
Cronbach's alpha	Continuous	The reported alpha coefficient for reliability
Other reliability estimate(s)	Continuous	Reliability estimate(s) other than Cronbach's alpha (e.g., McDonald's omega, test-retest coefficient)
Mean Age	Continuous	The average age of participants in the sample (in years)
SD of age	Continuous	The standard deviation of the participants' age in the sample
Gender distribution	Continuous	The proportion of women in the total sample size
Number of response categories	Continuous	Number of response categories employed when administering CCWS to the sample

The coders implemented the coding directly using the category names provided in Table 1. In studies with multiple samples that reported a separate alpha coefficient for each sample, a number was appended next to the author and publication details when coding the study tag (e.g., Stewart, 2021_1; Stewart, 2021_2; Stewart, 2021_3). As shown in Table 1, the numerical values specified in the original studies were used directly when coding continuous variables. Some adjustments were made to the categorical variables during data preparation for analysis, considering the number of studies in each subcategory. In the country title, the subcategories were coded as USA = (1), Turkey = (2), and a Europe subcategory was created by grouping other European countries, which were coded as (3). Countries (e.g., Vietnam, Israel) that could not be classified into the first three subcategories were coded as Others (4). After this change, the region name replaced the country name for the relevant variable. Given the limited number of studies in languages other than English and Turkish, the moderator analysis for the variable of scale language was conducted as English, Turkish, and other languages, coded as 1, 2, and 3, respectively.

### Data analysis

Vacha-Haase's (1998) meta-analytic RG was used in the data analysis. Only Cronbach's alpha was considered in the current investigation because the alpha coefficient was the most frequently used reliability index in individual studies. Prior to analysis, conversion was applied to the raw Cronbach's alpha values to normalize their negatively skewed distribution and stabilize the variance (Rodriguez and Maeda, 2006; Semma et al., 2018). Several methods (Fisher's *z*, Hakstian and Whalen's, and Bonett's transformation) have been introduced for transformation in RG meta-analysis. The most commonly used one in the studies has been Fisher *z*. However, this transformation is only suitable for correlation-based reliability coefficients (e.g., test–retest, parallel forms) (Sánchez-Meca et al., 2013). Since Cronbach's alpha is not a correlation coefficient, either Hakstian and Whalen's or Bonett's transformations should be employed in RG meta-analyses performed on the alpha coefficient (López-Ibáñez et al., 2024). Taking as reference the suggestion of Sánchez-Meca et al. (2013), Bonett (2010) 's formula was preferred for the transformation in this meta-analysis.

Along with the transformation method, another critical decision for the analysis involves selecting the appropriate model: fixed effects vs. random effects. The random effects model is generally the better choice because (a) it is more likely to align with the actual sampling distribution, (b) it does not impose a common effect size restriction, (c) it yields the same estimates as the fixed-effect model in the absence of heterogeneity, and (d) it enables the results to be generalized to a broader range of research than those included in the meta-analysis (Borenstein et al., 2010). Similarly, Field and Gillett (2010) argued that random effects are more realistic than the fixed-effect approach for real-world data in the social sciences and should be the norm in that context. In this regard, while calculating pooled alphas, the meta-analysis was conducted according to the random effects model, using the inverse variance method to weight the coefficients.

The heterogeneity among the studies was assessed using several statistics. The first statistic was the variance between studies (τ^2^), which was checked to see if it was different from zero. The restricted maximum likelihood method served as the estimator for the τ^2^ statistic. The second statistic was the Q-test, and its statistical significance served as evidence of heterogeneity. The third heterogeneity indicator analyzed was the *I*^2^ index. This index provides information about the magnitude of heterogeneity, unlike the Q-test, which produces a binary decision regarding the presence or absence of heterogeneity (Lin, 2019). Values of 75% or greater were interpreted as considerable heterogeneity when evaluating the *I*^2^ index (Deeks et al., 2008).

Due to heterogeneity, moderator analyses were conducted to identify the study descriptors and sample characteristics that may be sources of this heterogeneity. These analyses, performed for both continuous and categorical variables, utilized a mixed-effects model. The scale language and region variables, for which the number of primary studies in the subcategory was sufficient, were identified as categorical moderator variables. In contrast, the mean and standard deviation of age (in years), the female ratio in the sample, and the total number of items responded to by participants during the administration of the CCWS were included as continuous moderator variables in the meta-analysis. While meta-regression was executed for continuous moderator variables, an analog ANOVA was conducted for the categorical ones.

To audit the risk of publication bias, a funnel plot was created using Bonett's transformation values of Cronbach's alphas and standard errors. Additionally, statistical methods such as Rosenthal (1979)'s Fail-safe N, Begg and Mazumdar (1994)'s rank correlation, Egger's regression test (Egger et al., 1997), and Duval and Tweedie (2000)'s trim and fill were evaluated. The criterion for Rosenthal's Fail-safe N approach used the equation N/(5k+10), where *N* and *k* represent the number of missing studies and the number of studies included in the meta-analysis, respectively, and a value >1 indicates no publication bias (Rosenthal, 1979). Finally, both types of reliability induction—“by omission (i.e., failing to mention reliability throughout the manuscript)” and “by the report (i.e., presenting the reliability estimates of previous studies)” (Sánchez-Meca et al., 2021)—were considered while calculating the reliability induction rate. All statistical analyses were conducted with the meta package (Balduzzi et al., 2019) and the metafor package (Viechtbauer, 2010) in the R Studio environment.

## Results

Within the scope of the research, the characteristics of the studies included in the meta-analysis were examined first, and the findings obtained are displayed in Table 2. It is apparent from the table that the studies incorporated in this meta-analysis were published between 2020 and 2024. At this point, the question may arise: “If the CCWS was first published in 2021, how is it that the scale was used in an article in 2020?” In the study by Orr et al. (2020), the study that CCWS published was cited as “submitted for publication.” Therefore, the publication date of the relevant article is before the CCWS' publication year. A closer inspection of the table demonstrates that a single alpha coefficient was calculated in 35 of the 37 studies, while the studies by Stewart (2021) and Hamama-Raz and Shinan-Altman (2024) reported three and two separate Cronbach's alpha estimates, respectively. Table 2 also shows that the studies were conducted in different regions; nevertheless, most of them were based in Türkiye, followed by the US. Hence, the scale was predominantly applied to the Turkish and English languages. The table shows that the female ratio to the total sample size ranged from 0.324 to 1.00. Accordingly, among the studies using CCWS, one was conducted only with women, but none included only men. The sample size of the studies selected for analysis varied from 54 to 3,412, totaling 18,067 participants. The mean and standard deviation of the participants' ages were between 20.56 and 44.87 and 1.06 and 16.78, respectively. When the number of items answered by the participants (*k*) in the studies was examined, it was inferred that in some studies, only the CCWS was applied; thus, the number of items answered was 10, while in other studies, several instruments were administered to the sample simultaneously, and the number of items answered reached up to 140. Since a five-point rating scale was employed in all research included in the meta-analysis and almost all studies had a mixed composition concerning participants' education levels, no descriptions related to these variables were included in the table.

**Table 2:** Summary of the 37 included studies with 40 Cronbach's alpha estimates.

**Study tag**	**Cronbach's alpha**	**Sample size**	**Female ratio**	**Mean age**	**SD of age**	**Scale language**	**Region**	***k***
Orr et al. (2020)	0.95	1,100	0.665			English	US	35
Stewart (2021)_1	0.95	600	0.500	22.3	5.9	English	US	22
Stewart (2021)_2	0.90	54	0.833	20.9	1.06	English	US	10
Stewart (2021)_3	0.91	54	0.833	20.9	1.06	English	US	10
Gezer and Ilhan (2021)	0.90	233	0.734	22.87	4.03	Turkish	Türkiye	10
Özbay and Alci (2021)	0.98	308	0.536			Turkish	Türkiye	10
Aydemir (2022)	0.95	440	0.634			Turkish	Türkiye	23
Qi et al. (2022)	0.93	224	0.770	37.43	16.78	English	US	48
Aslan et al. (2023)	0.91	358	0.324	33.86	12.53	Turkish	Türkiye	10
Baykara Mat et al. (2023)	0.90	260	0.760			Turkish	Türkiye	21
Demir et al. (2023)	0.97	321	1.000	37.35	11.57	Turkish	Türkiye	140
Dogan and Buz (2024)	0.93	449	0.820			Turkish	Türkiye	20
Dugstad et al. (2023)	0.95	3,412				English	Europe	18
Ecer et al. (2023)	0.98	60	0.500	22.5		Turkish	Türkiye	16
Kars Fertelli (2023)	0.89	511	0.769	32.15	9.03	Turkish	Türkiye	42
Plohl et al. (2023)	0.96	442	0.758	21.57	1.67	Slovenian	Europe	94
Shinan-Altman and Hamama-Raz (2023)	0.94	402	0.515	41.63	14.89	English	Israel	25
Smith et al. (2023)	0.87	415	0.508	33.38	11.37	English	Mixed (46.5% from the US)	76
Türkarslan et al. (2023)	0.94	605	0.699	26.54	8.25	Turkish	Türkiye	72
Usta (2023)	0.92	651	0.599			Turkish	Türkiye	10
Ayar et al. (2024)	0.912	350	0.626	20.9	2.5	Turkish	Türkiye	30
Ceylan et al. (2024)	0.934	206	0.879			Turkish	Türkiye	31
Duckwitz (2024)	0.94	179	0.57	37.69	11.23	English	Europe	78
Duran and Kaynak (2024)	0.74	190	0.768	30.87	6.45	Turkish	Türkiye	21
Geraci et al. (2024)	0.87	224	0.610	21.04	1.65	Italian	Europe	74
Gottwlad (2024)	0.93	88	0.71			German	Europe	59
Hamama-Raz and Shinan-Altman (2024)_1	0.94	202	0.460	44.87	13.94	Hebrew	Israel	21
Hamama-Raz and Shinan-Altman (2024)_2	0.94	402				Hebrew	Israel	21
Ilaslan and Orak (2024)	0.90	289	0.768	20.56	1.51	Turkish	Türkiye	40
Kurt and Akdur (2024)	0.98	1229	0.688			Turkish	Türkiye	23
Larionow et al. (2024)	0.93	420	0.824	26.2	10.61	Polish	Europe	28
Le et al. (2024)	0.947	528	0.657			English	Vietnam	39
Pereira et al. (2024)	0.896	577	0.645	32.62	14.71	English	Europe	75
Regnoli et al. (2024)	0.91	283	0.517	21.3	1.7	Italian	Europe	50
Semenderoglu et al. (2024)	0.90	200	0.560			Turkish	Türkiye	10
Servan (2024)	0.86	582	0.488			Turkish	Türkiye	34
Shepherd et al. (2024)	0.91	442	0.821	32.45	12.5	French	Europe	90
Stewart et al. (2024)	0.95	308	0.802	21	2	English	US	41
Tümer et al. (2024)	0.91	211	0.754	21	1.52	Turkish	Türkiye	42
Türe (2024)	0.946	258	0.705	22.18	3.02	Turkish	Türkiye	27

**Figure 2:**
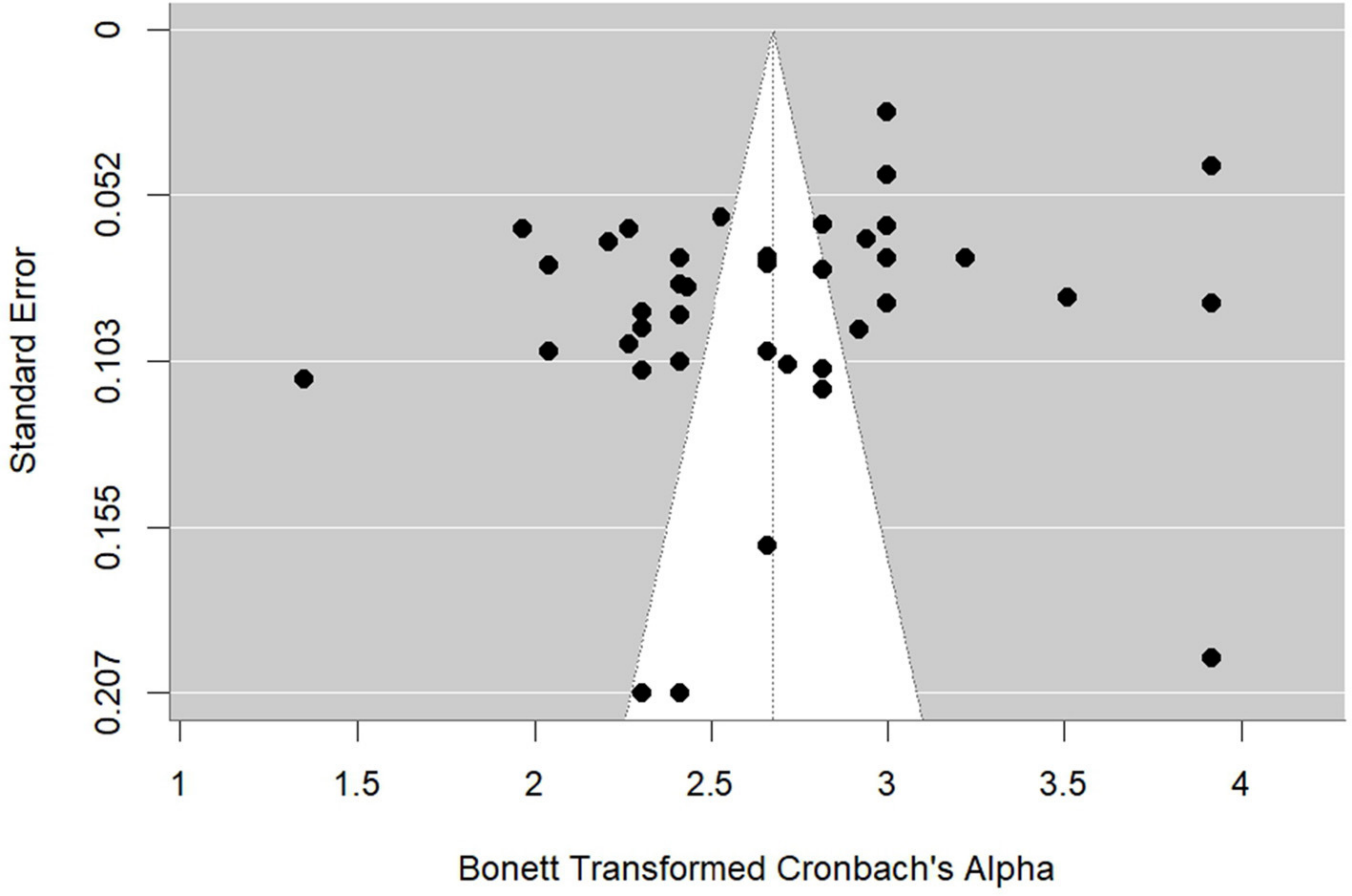
Funnel plot.

After examining the descriptive characteristics of the studies, publication bias was assessed. The funnel plot created for this purpose is shown in Figure 2.

Figure 2 shows that the funnel plot has a distribution close to symmetrical. This implies the absence of publication bias. However, merely examining the funnel plot is not sufficient to settle the question of publication bias. For this reason, along with the funnel plot, results based on statistical methods were also examined. Firstly, Duval and Tweedie's (2000) trim and fill method result was scrutinized, which is an approach based on a funnel plot. Accordingly, the number of studies to be added to the right of the plot to ensure full symmetry of the funnel plot is 9, indicating publication bias. When the results of the Rosenthal Fail-safe *N* method were examined, it was found that the number of missing studies required to render the average effect size statistically insignificant was 743,191. Since *N* = 743,191 and *k* = 40, the value of *N*/(5*k*+10) was >1, which asserted that there was no publication bias. Lastly, Begg and Mazumdar (1994)'s rank correlation and Egger's regression test (Egger et al., 1997) were explored. The z values of Kendall's τ and Egger's regression test were −0.107 and −0.524, respectively, and both were found to be statistically insignificant. Taken together, all bias statistics suggest that there is no publication bias. After concluding that there was no publication bias, the meta-analytic RG and heterogeneity results of the CCWS were exhibited in Table 3.

**Table 3:** Results of overall Cronbach's alpha and heterogeneity.

	***k***	***α [LLα- ULα]***	**Z**	***Q***	***I^2^***	***τ^2^*[SE]**
Overall Cronbach's α	40	0.932 [0.919–0.942]	32.164^*^	2,044.170^*^	98.12%	0.268 [0.06]

^*^p < 0.0001, *k*, Number of alpha coefficients; LLα, Lower Limit of Cronbach's α; ULα, Upper Limit of Cronbach's α.

**Figure 3:**
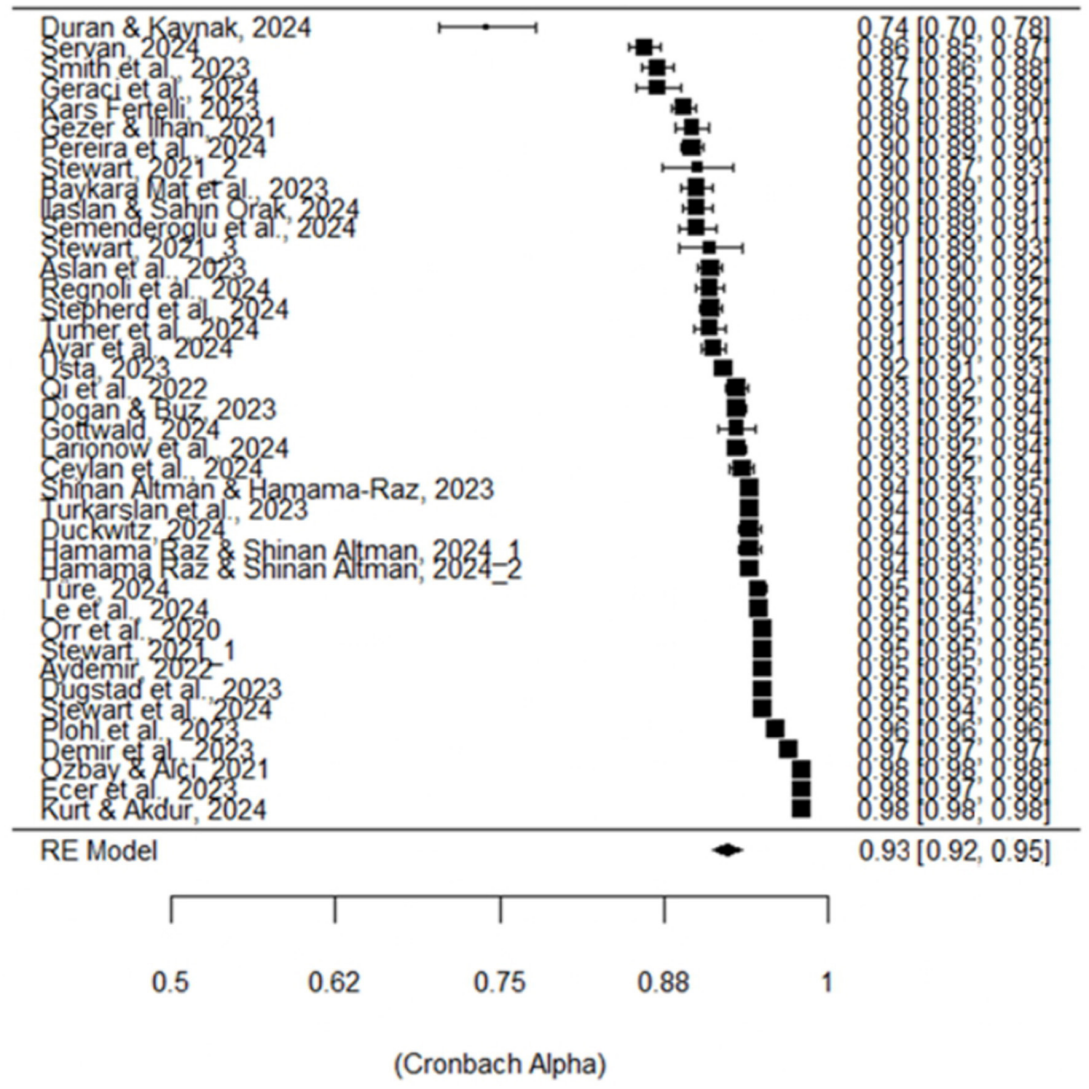
Forest plot.

According to Table 3, the statistically significant *Q*-test indicated heterogeneity among the reliability estimates. The *I*^2^ value (98.12%), which is >75%, indicated a high level of heterogeneity. The fact that the τ^2^ value (0.268) was significantly different from 0 provided further evidence of the variance among studies. Although it is a relatively subjective argument, the presence of heterogeneity can also be observed in the forest plot in Figure 3, where the lowest Cronbach's alpha coefficient was 0.74 (Duran and Kaynak, 2024), and the highest was 0.98 (Ecer et al., 2023). Overall, all evidence supported the existence of heterogeneity.

The pooled Cronbach's alpha reliability coefficient of the CCWS was found to be 0.932 [95% Confidence Interval (CI) = 0.919–0.942]. This coefficient was statistically significant and exceeded the widely accepted threshold of 0.70 for the reliability index. It was also very close to Cronbach's alpha value of 0.95, which was calculated by Stewart in his study developing the CCWS. Due to the high heterogeneity observed in the RG meta-analysis, it became essential to investigate the sources of this variance. Therefore, the variation in the Cronbach's alpha coefficient based on the moderator variables was assessed. Moderator analysis was primarily conducted for the categorical variables of region and scale language. Table 4 presents the analog ANOVA results applied for this purpose.

**Table 4:** Results of the analog ANOVA using categorical moderator variables as independent variables.

**Categories**		***k***	***α [LLα-ULα]***	**Z**	***Q***	***df***	***p***
Region	Türkiye	20	0.932 [0.914–0.947]	21.961^*^	0.216	3	0.975
	Europe	9	0.926 [0.895–0.948]	14.288^*^			
	USA	6	0.935 [0.899–0.958]	12.146^*^			
	Others	5	0.932 [0.890–0.958]	10.975^*^			
Scale language	English	12	0.932 [0.908–0.950]	17.169^*^	0.081	2	0.961
	Turkish	20	0.932 [0.914–0.946]	22.219^*^			
	Others	8	0.928 [0.895–0.950]	13.724^*^			

^*^p < 0.0001, *k*, Number of alpha coefficients; LLα, Lower Limit of Cronbach's α; ULα, Upper Limit of Cronbach's α; df, degree of freedom.

It is evident from Table 4 that Cronbach's alpha coefficients for all subcategories related to the regional variable exceeded 0.90 and were statistically significant. The lowest and highest overall alpha coefficients were recorded for Europe (0.926 [95% CI 0.895–0.948]) and the USA (0.935 [95% CI 0.899–0.958]), respectively. However, the differences observed between the pooled internal consistency estimates of Türkiye, Europe, the USA, and other regions were not statistically significant. A similar trend was observed for the scale language variable, where Cronbach's alpha values in all subcategories were >0.90 and were statistically significant. The pooled alpha values for both the English (0.932 [95% CI 0.908–0.950]) and Turkish (0.932 [95% CI 0.914–0.946]) versions of the CCWS were identical, while the overall alpha of other languages was lower (0.928 [95% CI 0.895–0.950]). Following the categorical variables, a continuous moderator analysis was performed using the mean age, standard deviation of age, female ratio, and the total number of items applied to the sample. Table 5 presents the meta-regression results with continuous moderator variables as predictors.

**Table 5:** Results of meta-regression using continuous moderator variables as predictors.

**Continuous moderator**	***k***	**β [LLβ-ULβ]**	**Z**	***p***	***Q_*E*_***	***R^2^ (%)***
Mean age	26	0.002 [−0.025–0.028]	0.138	0.890	680.399^*^	0.000
The standard deviation of age	25	0.009 [−0.025–0.043]	0.532	0.595	634.106^*^	0.000
Female ratio	38	−0.051 [−1.293–1.190]	−0.081	0.935	1,940.336^*^	0.000
Number of total items	40	0.003 [−0.005–0.006]	0.222	0.824	2,013.923^*^	0.000

^*^p < 0.0001, *k*, Number of alpha coefficients; LLβ, Lower limit of the slope; ULβ, Upper limit of the slope; *Q*_*E*_, *Q* values for residuals; *R*^2^, explained variances.

As shown in Table 5, the slopes of the continuous variables were not statistically significant. Based on this result, we can conclude that the changes in the analyzed continuous variables did not produce a statistically significant difference in Cronbach's alphas. Furthermore, the variance explained by these variables was found to be 0.000%. Conversely, the fact that all *Q*_*E*_ residual variance values were statistically significant indicates that the variability in Cronbach's alpha values can be attributed to other variables.

Eventually, the reliability induction rate was calculated. From Figure 1, it can be observed that one primary study could not be included in the meta-analysis due to omission. Conversely, the number of studies that induced reliability from prior research was 14. In this context, since CCWS was applied in 51 studies, the induction rate was 29.41%. When the reliability induction through omission and reporting was calculated separately, it was determined that these values were 1.96% and 27.45%, respectively.

## Discussion and conclusion

Among the various psychological impacts of climate change, the ones that empirical research has primarily focused on are eco-anxiety and worry (Regnoli et al., 2024). Despite the growing interest in anxiety and worry related to climate change, relatively few validated scales address the structures involved (Plohl et al., 2023). A promising new instrument for assessing climate change worry is the CCWS (Larionow et al., 2024; Shepherd et al., 2024). This tool, which evaluates disturbing thoughts about climate change through 10 items, was initially developed by Stewart (2021). After the development of its original version in English, the CCWS was adapted into Turkish (Gezer and Ilhan, 2021; Özbay and Alci, 2021), Italian (Donati et al., 2025; Innocenti et al., 2022), Polish (Larionow et al., 2024), Slovenian (Plohl et al., 2023), and French (Shepherd et al., 2024) and has been used in many studies. The published research has provided substantial information regarding the reliability of the measures obtained from the CCWS, but it remains at the individual level. Therefore, a study designed to provide more comprehensive evidence about the scale's reliability was deemed important, leading to the present RG meta-analysis.

This study combined the Cronbach's alpha coefficients calculated in individual studies utilizing the CCWS in a statistically rigorous manner and tested various moderator variables that could be effective in estimating differing internal consistency coefficients for scale scores from one administration to another. When examining the descriptive properties of the analyzed studies, it was found that more than half of them were conducted with a Turkish sample. The CCWS was adapted into Turkish in the same year its original English form was published. The fact that the CCWS has been deployed in Turkey for about 4 years may have contributed to the increased number of studies conducted with the Turkish sample compared to the cultures to which it was adapted later. An additional factor influencing the extensive use of CCWS in the Turkish sample may be that Türkiye is located in a geographical area highly susceptible to climate change (Sen, 2013), which could prompt research focused on worry about climate change.

It was found that Cronbach's alpha coefficients for the measurements obtained from the CCWS in individual studies ranged from 0.74 to 0.98; however, in most cases, they were 0.90 or greater. This finding indicates that the CCWS generally produced measures with analogous internal consistency, although some individual studies had Cronbach's alpha estimates that were significantly divergent. The RG meta-analysis revealed a pooled Cronbach's alpha of 0.932 for the CCWS. Hogan (2019) describes reliability coefficients exceeding 0.90 as excellent and further states that measurements with this level of reliability can be used to make critical decisions about individuals, such as placement on an occupational licensing examination or diagnosing someone as mentally impaired in a forensic case. Therefore, the measurements yielded from CCWS demonstrate very high reliability, and there is strong agreement among the items of the scale.

The findings from meta-regression and analog ANOVA indicated that none of the examined variables moderated the psychometric properties of the CCWS. This result suggests that the CCWS is a robust measurement tool that is not influenced by potential confounders. To clarify, the CCWS produces scores with high internal consistency regardless of language, geographical location, age, gender, or the total number of items answered during the administration. The fact that gender and age were not significant moderators of the internal reliability of the CCWS aligns with the findings of the study conducted by Donati et al. (2025), which verified the measurement invariance of the CCWS across biological sex and age groups. Conversely, the results regarding the moderator variable of the total number of items answered were somewhat contrary to expectations. When participants respond to a large number of items, factors such as fatigue and inattention may decrease the reliability of the data. However, the results of the moderator analysis revealed that even when participants answered an excessive number of items due to the simultaneous use of the CCWS with other instruments, the reliability coefficient remained as high as when the scale was administered alone. Another important finding from the moderator analysis worth highlighting is the absence of region- and language-related differences in the reliability of CCWS scores, supporting the fact that adaptation studies for the scale have generally yielded similar results to their original form. It is certainly possible that more than half of the studies included in the meta-analysis were conducted in Türkiye using the Turkish version of the CCWS, which may have contributed to this outcome.

The results indicate that the addressed moderator variables did not affect the internal consistency of the CCWS, which could be attributed to various factors. Firstly, climate change is a global issue that can generate similar worries in all individuals, regardless of their demographics. In other words, people of all ages, genders, and geographic backgrounds may worry equally about the effects of climate change, resulting in minimal variability in the internal reliability of the CCWS across the listed variables. Another possible explanation for the moderator variables being non-significant predictors of the CCWS's reliability is that the scale was designed to capture broad, collective, and universally shared concerns that extend beyond age, gender-based perceptions, or culturally specific thoughts. In summary, the moderator analysis results indicate that factors related to the administration conditions of the scale and/or other moderators beyond the variables examined in this study may have contributed to the variability of the CCWS score reliability across the primary studies.

The study also aimed to determine the ratio of reliability induction in the research utilizing the CCWS. It was found that 29.41% of the studies included in the meta-analysis exhibited reliability induction, either by reporting a reliability estimate from previous applications of the scale or by not mentioning reliability at all. This percentage was similar to the rates of reliability induction reported by López-Nicolás et al. (2021), Vicent et al. (2019), and Demir and Demircioglu (2024) in their RG meta-analysis studies. Conversely, it was considerably lower than the average reliability induction rate of 78.6% reported by Sánchez-Meca et al. (2015), who systematically reviewed RG meta-analysis research (cited in López-Nicolás et al., 2021), the 67.95% reported by Correia et al. (2025) in their manuscript on the RG of the pre-sleep arousals scale, and the 76.10% found by Alcocer-Bruno et al. (2020) in the RG of the Medical Outcome Study-HIV Health Survey. As the increase in RG meta-analyses has heightened researchers' awareness of the need to report reliability estimates with the available data rather than inferring them from prior research (López-Nicolás et al., 2021), the relatively low-reliability induction rate observed for the CCWS may be due to the scale being relatively new. Indeed, Sánchez-Meca et al. (2015) identified a positive correlation between the proportion of reliability reporting and the publication year of 100 RG meta-analyses (cited in López-Nicolás et al., 2021). Nevertheless, it should also be noted that the reliability induction rate established in the current study remains a significant finding. It is hoped that this article, which addresses reliability induction as a sub-issue, will act as a caution for researchers planning to utilize the CCWS in the future and will contribute to further decreasing the reliability induction of the scale.

Consequently, CCWS scores demonstrated excellent internal consistency regardless of sample and study characteristics. Given this favorable result, it can be said that CCWS accurately measures worry about climate change, and researchers can confidently utilize the scale. Moreover, the fact that the CCWS can produce highly reliable scores with only 10 items shows that it can be administered quickly and is a powerful instrument even for large survey studies, promoting its continued use. However, researchers should be cautious about the following matter when employing the scale: the high heterogeneity discovered in the reliability of CCWS scores among primary studies confirmed that its reliability cannot be generalized across different population characteristics and study conditions and that inducing reliability is an approach that needs to be eradicated. The variation in the reliability of CCWS from one study to another indicates that when presenting the reliability analysis results, the sample and study characteristics under which reliability was calculated should also be described in detail.

## Limitations and future directions

The current study has certain limitations that suggest several areas for future research. Firstly, this RG meta-analysis was confined to Cronbach's alpha coefficient; thus, the reliability considered here pertains only to internal consistency. Because test-retest reliability, a measure of stability, and omega, another internal consistency coefficient, were rarely reported in the available studies employing the CCWS, these coefficients were excluded from the current RG research. In light of this limitation, it is recommended that RG meta-analyses also consider these coefficients, as the number of studies reporting the test-retest reliability and omega coefficient of CCWS scores increases in the future. Furthermore, future RG meta-analyses conducted on the CCWS can explore whether variables beyond the moderators examined in the current study influence the reliability estimates. Finally, as studies using the CCWS and reporting factor loadings of its items or inter-item correlation matrices increase in the literature, meta-analytic factor analysis research can also be pursued.

## Data Availability

Publicly available datasets were analyzed in this study. This data can be found here: http://bit.ly/4i8j9LT.
